# *Brucella melitensis omp*31 Mutant Is Attenuated and Confers Protection Against Virulent *Brucella melitensis *Challenge in BALB/c Mice

**DOI:** 10.4014/jmb.1908.08056

**Published:** 2020-01-17

**Authors:** L Verdiguel-Fernández, R Oropeza-Navarro, Adolfo Ortiz, MG Robles-Pesina, J Ramírez-Lezama, A Castañeda-Ramírez, A Verdugo-Rodríguez

**Affiliations:** 1 Laboratorio de Microbiología Molecular, Departamento de Microbiología e Inmunología, Facultad de Medicina Veterinaria y Zootecnia, Universidad Nacional Autónoma de México, Avenida Universidad 3000, colonia UNAM CU, Coyoacán C.P 04510, CdMx, México; 2 Departamento de Microbiología Molecular, Instituto de Biotecnología, Universidad Nacional Autónoma de México, Cuernavaca, Morelos, México; 3 Unidad de Bioseguridad de Brucella, Departamento de Microbiología e Inmunología, Facultad de Medicina Veterinaria y Zootecnia, Universidad Nacional Autónoma de México, Coyoacán, México; 4 Centro Nacional de Servicios de Diagnóstico en Salud Animal (CENASA), Servicio Nacional de Sanidad, Inocuidad y Calidad Agroalimentaria, Tecámac, México; 5 Departamento de Patología, Facultad de Medicina Veterinaria y Zootecnia, Universidad Nacional Autónoma de México, Coyoacán, CdMx, México; 6 Departamento de Zootecnia, Universidad Autónoma de Chapingo, Texcoco, México

**Keywords:** *Brucella melitensis*, *omp*31, virulence, vaccines, immunity

## Abstract

For control of brucellosis in small ruminants, attenuated *B. melitensis* Rev1 is used but it can be virulent for animals and human. Based on these aspects, it is essential to identify potential immunogens to avoid these problems in prevention of brucellosis. The majority of OMPs in the Omp25/31 family have been studied because these proteins are relevant in maintaining the integrity of the outer membrane but their implication in the virulence of the different species of this genus is not clearly described. Therefore, in this work we studied the role of Omp31 on virulence by determining the residual virulence and detecting lesions in spleen and testis of mice inoculated with the *B. melitensis* LVM31 mutant strain. In addition, we evaluated the conferred protection in mice immunized with the mutant strain against the challenge with the *B. melitensis* Bm133 virulent strain. Our results showed that the mutation of *omp*31 caused a decrease in splenic colonization without generating apparent lesions or histopathological changes apparent in both organs in comparison with the control strains and that the mutant strain conferred similar protection as the *B. melitensis* Rev1 vaccine strain against the challenge with *B. melitensis* Bm133 virulent strain. These results allow us to conclude that Omp31 plays an important role on the virulence of *B. melitensis* in the murine model, and due to the attenuation shown by the strain, it could be considered a vaccine candidate for the prevention of goat brucellosis.

## Introduction

The microorganisms of the genus *Brucella* are facultative intracellular pathogens that infect a great variety of mammals, producing epizootic abortion in animals and a febrile septicemic disease in humans called Malta fever or undulant fever, one of the zoonotic diseases of worldwide importance [1]. Currently, ten species of the genus *Brucella* have been recognized, although *B. melitensis* is the most isolated and virulent species in humans while also being the etiological agent of goat brucellosis, which causes heavy economic losses as it leads to infertility in males, abortions and mastitis in females, and arthritis in animals [2]. Therefore, vaccination has to be considered the fundamental tool to block the spread of brucellosis among animals. *B. melitensis* Rev1 vaccine is recognized as the most effective vaccine strain for the control and prevention of goat and sheep brucellosis, however, its application has adverse effects since it can cause abortions when pregnant animals are vaccinated. Furthermore, lactating females can secrete the vaccine strain through milk, infecting others animals; the vaccine also interferes with serological diagnosis, besides being virulent for humans [3-5]. *Brucella* spp. are short rods, gram negative, immobile, not sporulated and without capsule, so this pathogen does not have classic virulence factors like other bacteria. Based on these findings, several studies have concluded that many aspects of *Brucella* virulence are related to the characteristics of its outer membrane (OM). OM of *Brucella* has particular properties: it is resistant to the action of cationic peptides (lysozyme, lactoferrin, defensins or cathelicidins), detergents and non-immune serum [6-8]. The major components of *Brucella* OM are lipopolysaccharide (LPS) and outer membrane proteins (OMPs). OMPs are exposed on the surface of the bacteria and come into direct contact with cells and effectors of the immune response of host organism [9]. Therefore, the study on this field is of great interest, since it can provide a broader knowledge about the mechanisms of host-parasite interaction used by this genus, which could also allow the development of new attenuated vaccines better than the existing ones [10, 11]. In recent years, several investigations have been carried out in order to determine the influence of the Omp25/Omp31 family on virulence of *Brucella* [12]. Regarding Omp31, several studies have shown that it is about 32% identical with HbpA, which belongs to the hemin-binding protein (Hbp) family of *Bartonella quintana* [13]. In this respect, several studies have also been performed to verify that Omp31 of *B. suis* possesses some capacity to bind hemin, and that the expression of the gene that encodes it, is induced when *B. suis* is grown under limited iron conditions [14]. It is also known that Omp31 is not necessary for the virulence of *B. abortus*, since in this species the gene coding for it is not present due to a deletion in its genome [15, 16]. Besides, it has been shown that virulence of *B. melitensis* Rev1 vaccine strain in mice is not affected after *omp*31 deletion [17], although it should be noted that Rev.1 strain has a lower level of *omp*31 expression than that present in the *B. melitensis* 16M strain under laboratory conditions, and this fact can only be related to strain attenuation [18]. Other reports showed that the absence of this protein in the outer membrane of the *B. ovis* PA strain, although it reduces by one logarithm the maximum levels of splenic colonization of the bacteria in mice, its persistence in spleen is not diminished [8]. In a recent study, Verdiguel *et al*. 2017 demonstrated that the mutation of *omp*31 altered the outer membrane properties of *B. melitensis* and caused a significant decrease in the internalization, survival and intracellular replication of the bacterium in murine macrophages J774.A1 and in HeLa cells [19], thus suggesting Omp31 could play a relevant role in the virulence of the bacteria. To verify this hypothesis, we aimed in this work to determine the effect of *omp*31 mutation on *B. melitensis* virulence in a murine model and evaluate the protection conferred in mice immunized with the mutant strain.

## Materials and Methods

### Bacterial Strains and Growth Conditions

The strains used in this work are listed in Table 1. *Brucella* strains were cultured at 37°C in a 5% CO_2_ atmosphere for 72 h. They were typically propagated in *Brucella* broth (BB; Difco Laboratories, USA) or *Brucella* agar (BA; Difco Laboratories), both supplemented with 0.3% yeast extract (YE; Difco Laboratories) and 5% fetal bovine serum (FBS; GIBCO-BRL Life Technologies, Germany) (BB-YE-FBS and BA-YE-BFS). Prior to use, the strains were characterized by PCR and biochemical tests for the identification of microorganisms of the genus *Brucella* as well as Triple Sugar Iron (TSI), citrate, urea, hydrogen sulfide-indole-motility (SIM)), and Gram stain. *Brucella* were provided by MML (Molecular Microbiology Laboratory, Immunology and Microbiology Department, FMVZ-UNAM, Mexico). *B. melitensis* LVM31 was constructed by Verdiguel *et al.* [19].

### Experimental Animals and Ethical Guidelines

Corresponding groups are listed in Tables 2, 3, and 4. Representative groups of mice were required so that blood sampling and organ harvesting could be done at different times in order to evaluate residual virulence and the protection conferred by the mutant strain in comparison with the vaccine strain in order to perform the statistical analysis of the results. Mice were donated by the National Center for Diagnostic Services in Animal Health (CENASA).

Every day the health and behavior of mice were evaluated, while signs of any infectious disease were investigated. If the animals presented any alteration of the aforementioned parameters, they were euthanized. Mice were euthanized by cervical dislocation according to NOM-062-ZOO-1999 (Mexico) and the carcasses were incinerated according to NOM-087-ECOL-SSA1-2002 (Mexico).

Once the animals were euthanized, the spleen was removed under aseptic conditions. Each spleen was homogenized in sterile bags with 0.5 ml of PBS, serial dilutions were then made (v/v) and 20 μl of each homogenate was plated in triplicate and by double repetition per mouse on supplemented *Brucella* agar. Plates were incubated at 37°C under an atmosphere of 5% CO_2_ until the CFU count could be made [20]. This methodology was carried out to determine the residual virulence and protection conferred in mice inoculated with *Brucella* strains.

### Residual Virulence Determination in Mice Inoculated with *Brucella* Strains

Groups of 10 female and 10 male BALB/c mice of 8 weeks in age were inoculated intraperitoneally with approximately 5 × 10^5^ CFU of *B. melitensis* wild type, *B. melitensis* Rev1 vaccine and with *B. melitensis* LVM31 mutant strains suspended in 0.2ml of PBS. A negative control group of 10 female and 10 male BALB/c mice was inoculated with 0.2 ml of PBS (Table 2). Counts of CFUs from the spleen of 2 female mice per strain were determined at 3, 6, 9, 12, and 15 weeks post inoculation, while counts of CFUs from the spleen of 2 male mice per strain were determined at 4, 8, and 12 weeks post inoculation.

### Evaluation of Conferred Protection of Mice Immunized with Mutant and Vaccine Strains against Challenge with the Virulent *B. melitensis* Bm133 Strain

Groups of 10 8-week-old female BALB/c mice were immunized subcutaneously with 1 × 10^9^ CFU of *B. melitensis* LVM31 mutant and *B. melitensis* Rev1 vaccine strains in a 0.2 ml suspension [21, 22]. A control group of 10 unvaccinated mice was inoculated with 0.2 ml of PBS (Table 3). Thirty days after immunization mice were challenged intraperitoneally with 10^6^ CFU/mouse of the *B. melitensis* Bm133 wild-type strain[23]. Two mice were euthanized by cervical dislocation at weeks three, six and nine after challenge and spleens were collected from the mice and processed individually to quantify the CFUs per mouse.

### Evaluation of Residual Virulence in Pups Born of Females Inoculated with *Brucella* Strains

Groups of 3 female BALB/c mice of 10 weeks of age were inoculated intraperitoneally with a 0.2 ml suspension (5× 10^5^ CFU) of the wild-type, vaccine, and mutant strains or with 0.2 ml of PBS (Table 3). Seven days post inoculation; a male BALB/c mouse was introduced into the cages corresponding to each group. The male mouse was removed when the pregnant females started giving birth to avoid cannibalistic behavior. The offspring was separated three weeks after birth and confined in a different cage. At six weeks of age, they were euthanized and random samples of spleen were taken from three mice per group; spleens were processed as mentioned in methodology to determine splenic colonization of the offspring by CFU counting.

### Evaluation of Organ Lesions of BALB/c Mice Inoculated with *Brucella* Strains

At week 15 post infection, two mice were euthanized from each group of animals to take samples of spleen and testicles if a mouse was male; the tissues were fixed in 10% formalin (pH 7.2) and stained with hematoxylin and eosin (H & E); finally they were analyzed in a photonic force microscope to look for histopathological lesions [24].

### Statistical Analysis

Statistical analysis was performed using GraphPad Prism 7.0 (GraphPad software, USA). The data from assays for bacterial colonization in BALB/c mice at different time points and from efficacy studies were expressed as the mean log CFU ± SD for each group and analyzed by Student’s *t* test. *P* values of < 0.05 (*) and *p* values of < 0.01(**) were considered statistically significant.

## Results

### Residual Virulence Determination in Mice Inoculated with *Brucella* Strains

We evaluated the role of Omp31 on *Brucella* virulence in a murine model. Therefore, BALB/c mice were inoculated with *B. melitensis* LVM31 mutant strain in order to evaluate the residual virulence by determining the splenic colonization in comparison with mice inoculated with *B. melitensis* Bm133 virulent strain and *B. melitensis* Rev1 vaccine strain.

**Fig. 1:**
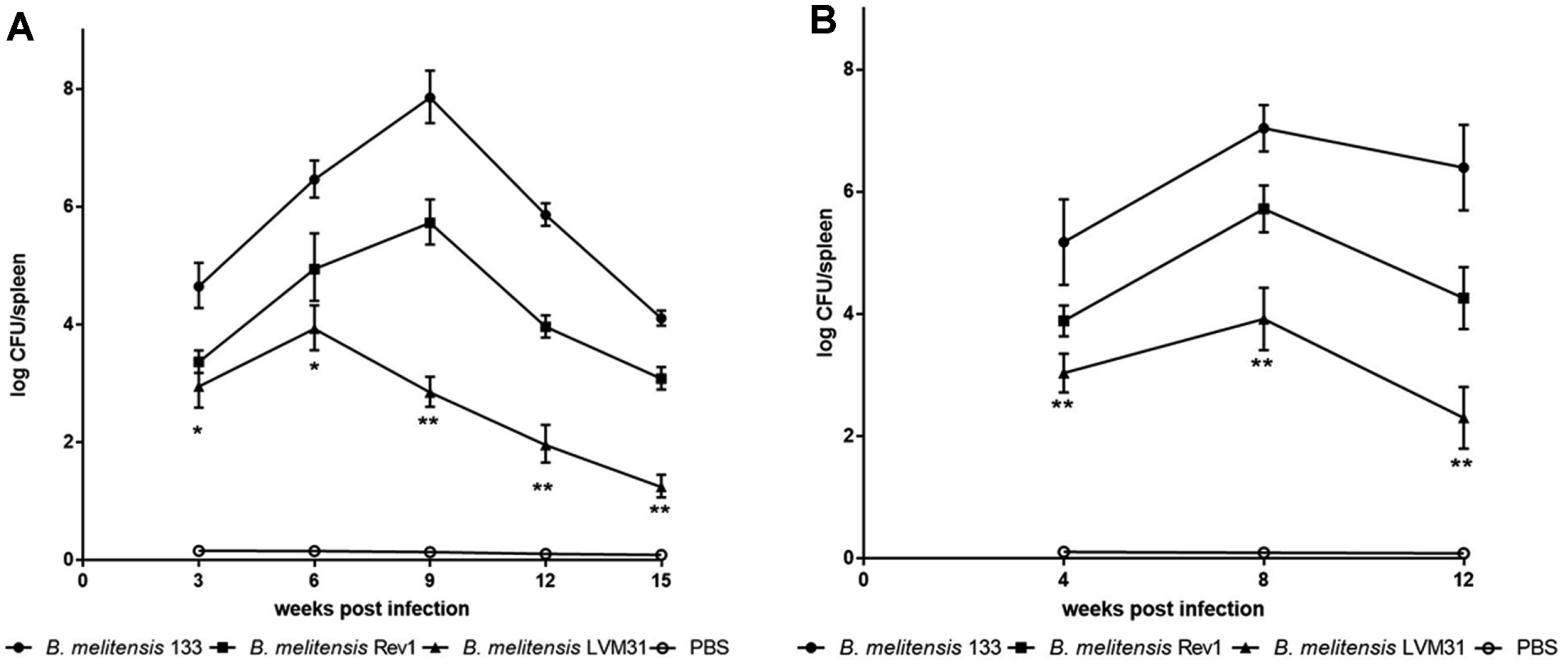
Residual virulence determination in mice inoculated with *Brucella* strains. (**A**) Splenic colonization of BALB/c female mice inoculated with *Brucella* strains. (**B**) Splenic colonization of male BALB/c mice inoculated with *Brucella* strains. Significant differences between LVM31 mutant strain and Bm133 are indicated by *(*p* < 0.05), **(*p* < 0.01).

The results of this work showed that there was a statistically significant decrease (*p* < 0.01) in splenic colonization in female mice inoculated with *B. melitensis* LVM31 mutant strain at 3, 6, 9, 12, and 15 weeks post infection compared with *B. melitensis* Bm133 wild-type strain, whereas in comparison with *B. melitensis* Rev1 vaccine strain there was a statistically significant decrease (*p* < 0.01) in the splenic colonization at 9, 12, and 15 weeks post infection (Fig. 1A).

Regarding male mice, it was shown that there was a statistically significant decrease (*p* < 0.01) in splenic colonization in mice inoculated with *B. melitensis* LVM31 mutant strain at 4, 8, and 12 weeks post infection compared to *B. melitensis* Bm133 virulent strain and with *B. melitensis* Rev1 vaccine strain (Fig. 1B). In both assays, the mice inoculated with 0.2 ml of PBS (negative control) had no colony growth in the culture media.

Based on these findings, we were able to verify the hypothesis that the Omp31 plays an important role in the virulence of *B. melitensis* in the murine model.

### Evaluation of the Protection Conferred in Mice Immunized with Vaccine Strains

The objective of this experiment was to determine if *B. melitensis* LVM31 mutant strain was able to protect female BALB/c mice against challenge of *B. melitensis* Bm133 wild-type strain. Mice immunized subcutaneously with 0.2 ml (1× 10^9^ CFU/ml) of LVM31 mutant strain, the Rev1 vaccine strain (positive control) or with 0.2 ml of sterile PBS (negative control), were challenged 30 days after vaccination with the virulent Bm133 strain. Spleen samples were taken from two mice per group and splenic colonization was determined at 3, 6, and 9 weeks post challenge. The CFU count was performed in triplicate and by repetition. The results represent the mean ± standard deviation of the log CFU/ml.

**Fig. 2:**
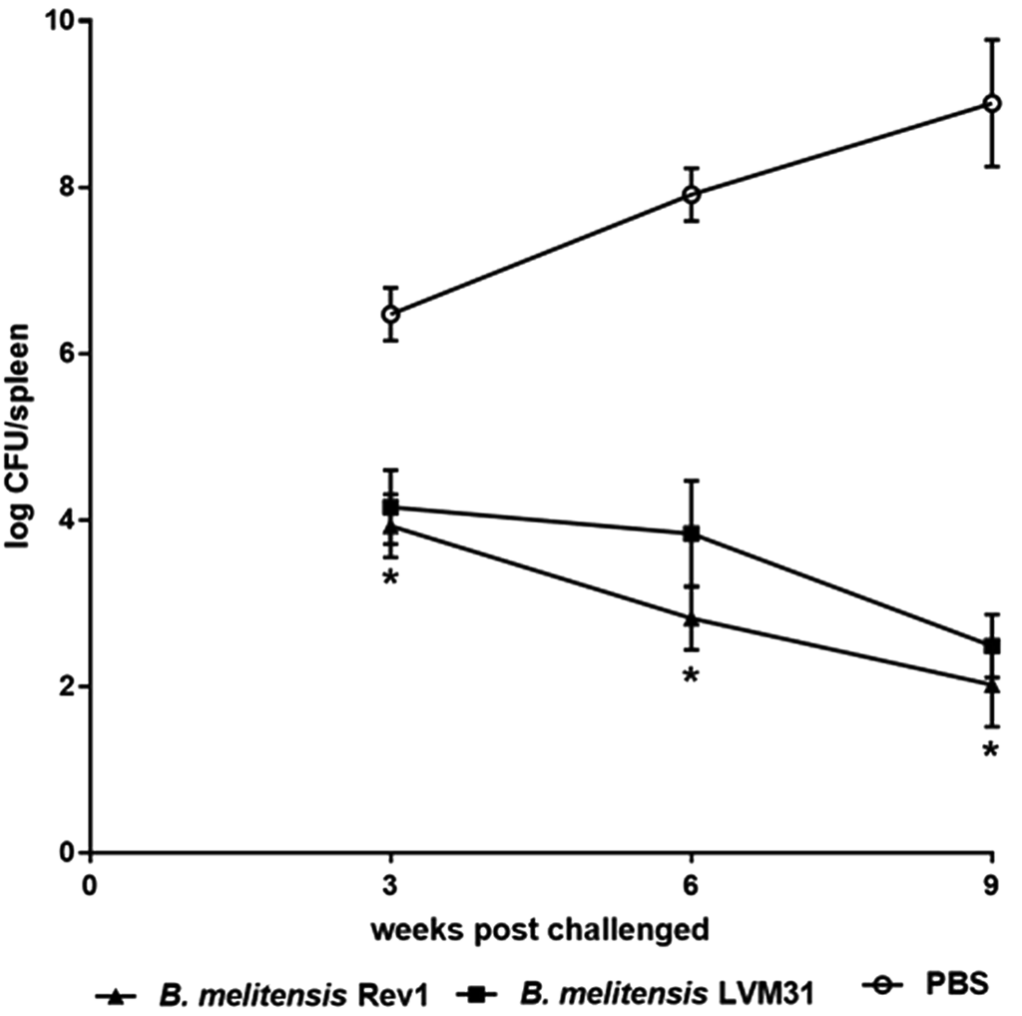
Evaluation of the conferred protection of female Balb/c mice immunized with LVM31 mutant strain and Rev1 vaccine strain against the challenge with the virulent *B. melitensis* Bm133 strain. The asterisks indicate significant differences between mice immunized with PBS and mice immunized with LVM31 mutant strain (*p < 0.05).

The results of this work showed that there was a decrease in splenic colonization in mice immunized with the vaccine strain compared to mice immunized with the mutant strain. Because there was no statistically significant difference between these strains, these results allow us to conclude that the mutant strain of *B. melitensis* LVM31 conferred a protection similar to the *B. melitensis* Rev1 vaccine strain against the challenge with the virulent *B. melitensis* Bm133 strain (Fig. 2).

### Evaluation of Residual Virulence in Pups Born of Females Inoculated with *Brucella* Strains

The objective of this experiment was to determine splenic colonization in BALB/c six-week-old mice born from BALB/c females inoculated with *Brucella* strains or PBS.

The results obtained in this work showed a statistically significant decline (*p* <0.05) in splenic colonization on mice born from females inoculated with *B. melitensis* LVM31 mutant strain compared to females inoculated with

**Fig. 3:**
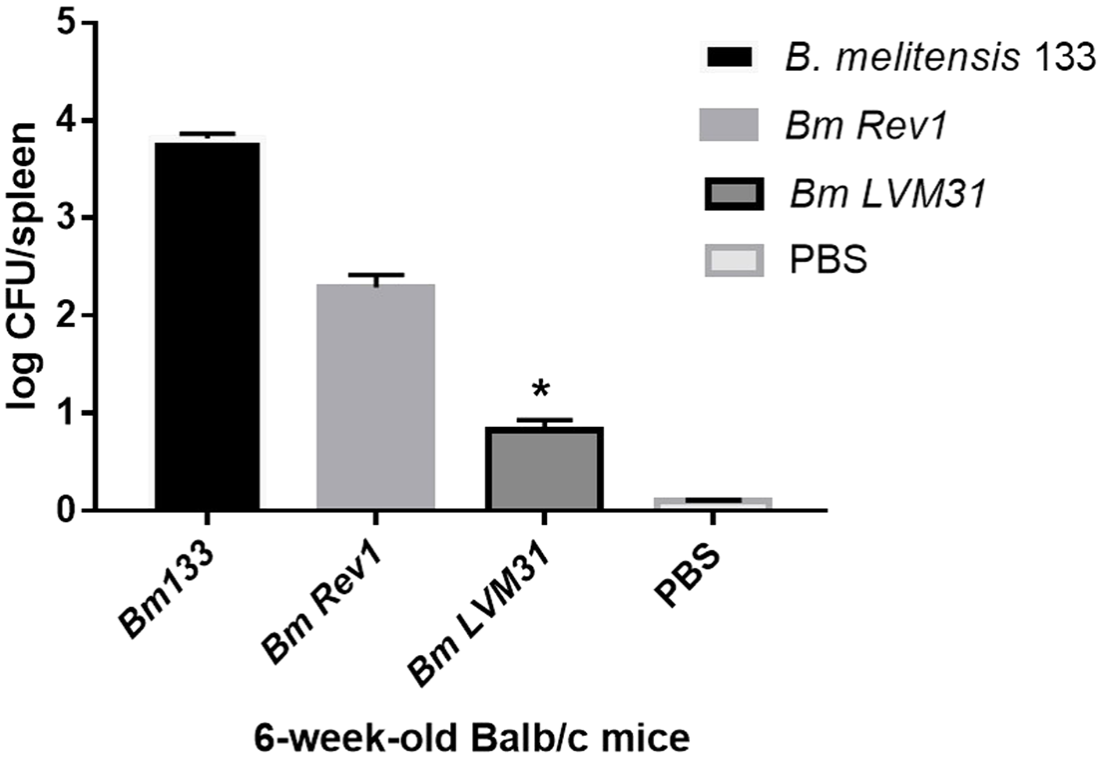
Evaluation of the virulence of offspring born of females inoculated with strains of *Brucella* spp. Significant difference between LVM31 mutant strain and Bm133 strain is indicated by *(*p* < 0.05).

Rev1 vaccine strain and Bm133 wild-type strain. Based on these results it can be concluded that the transmission of the bacteria from the mothers to the offspring was statistically significantly reduced in the mice inoculated with the LVM31 mutant strain in comparison with Rev1 and Bm133 strains (Fig. 3). It is important to clarify that with these results, we can not determine if the mechanisms of propagation of the bacteria were by vertical or horizontal transmission. Therefore, other studies will have to be carried out to determine the transmission mechanism.

### Determination of Organ Lesions of BALB/c Mice Inoculated with *Brucella* Strains

After demonstrating that the mutation of *omp*31 generated a decrease in splenic colonization as a consequence of attenuation in the virulence of the bacterium on murine model, samples of spleen and testicles (in case of males) of two mice were taken from each group 15 weeks post inoculation with *Brucella* strains in order to determine histopathological lesions of the organs.

**Fig. 4:**
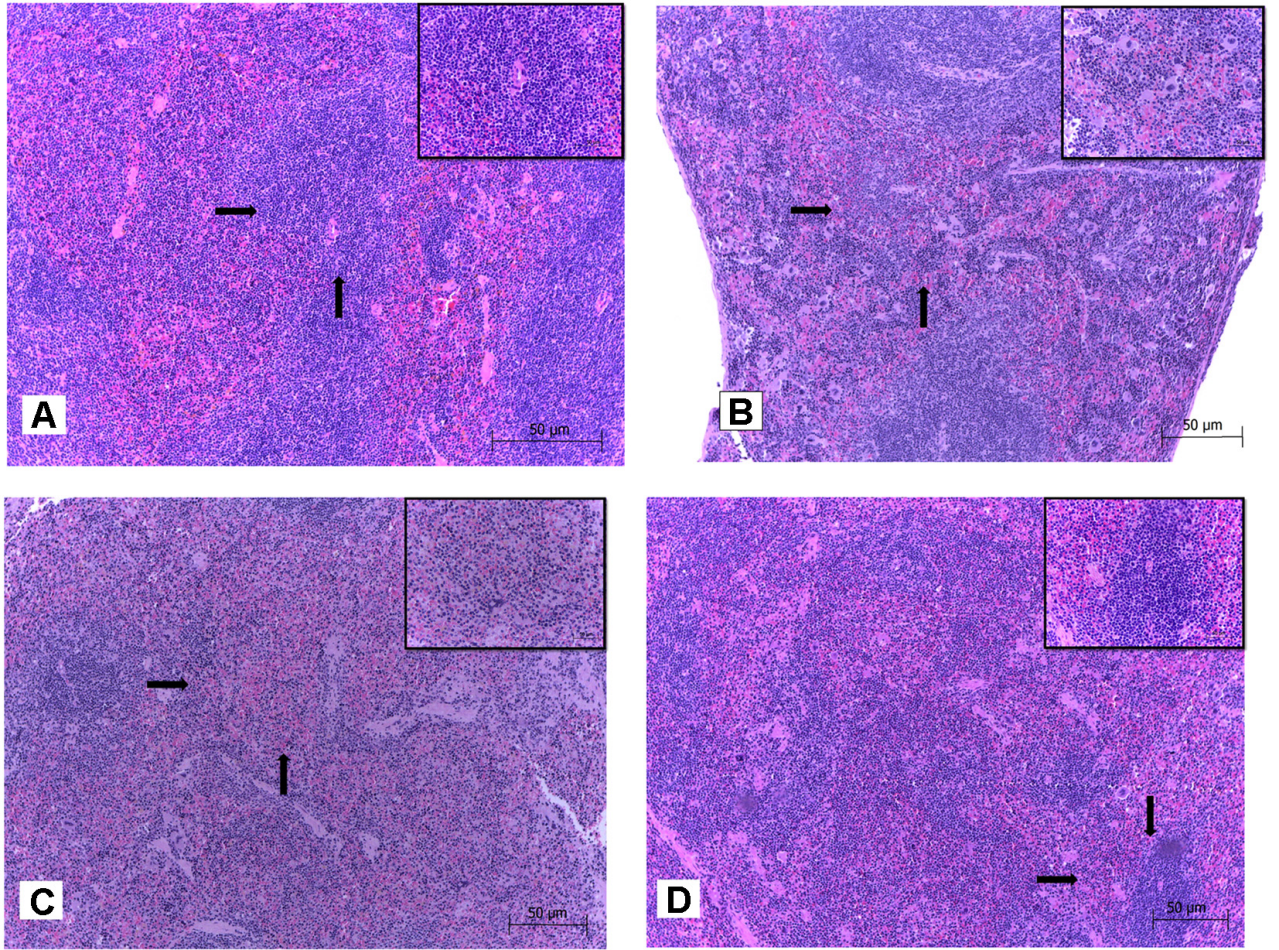
Spleen samples of mice inoculated with Brucella strains. (**A**) Mouse spleen inoculated with *B. melitensis* LVM31, (arrows point to spleen section showing organization of lymphoid follicles without apparent histopathological changes). (**B**) Mouse spleen inoculated with *B. melitensis* Rev1 (arrows point to spleen section showing disorganization of the lymphoid follicles; area in the box indicates diffuse moderate atrophy of lymphoid cells in white pulp with few megakaryocytes. (**C**) Mouse spleen inoculated with *B. melitensis* Bm133 (arrows point to spleen section showing discrete disorganization of the lymphoid follicles; in the box there is discrete atrophy of lymphoid cells in white pulp and no presence of megakaryocytes). (**D**) Mouse spleen inoculated with PBS (arrows point to spleen section showing organization of lymphoid follicles without apparent histopathological changes).

The results of this analysis revealed that the spleens of mice inoculated with PBS (Fig. 4D) and with *B. melitensis* LVM31 mutant strain (Fig. 4A) showed no apparent histopathological changes while the spleens of mice inoculated with vaccine strain (Fig. 4B) and wild-type strain (Fig. 4C) showed diffuse discrete lymphoid atrophy in white pulp with moderate megakaryocytes in red pulp and diffuse moderate lymphoid atrophy with abundant megakaryocytes, respectively.

On testes of mice inoculated with all *Brucella* strains, there were no apparent histopathological changes in any of the cases (data not shown).

## Discussion

*Brucella* is a facultative intracellular pathogen. As a particular feature, smooth *Brucella* species can invade, survive and multiply in professional phagocytic cells such as macrophages and dendritic cells, as well as non-phagocytic cells such as trophoblast cells and epithelial cells (HeLa cells) [25, 26]. Interestingly, *Brucella* spp. do not have classical virulence factors like other bacteria and several investigations have focused on identifying the role of the Omp25/Omp31 family in virulence of the genus *Brucella* [27]. The survival and replication of *Brucella* within macrophages, which represents the main host cells for this pathogen, will provide this microorganism protection against the complement and antibodies during their dissemination by the host, and also the ability to maintain themselves for long periods of time in the organ affected, with the consequent chronicity of the infection [28]. This feature of *Brucella* strains in the smooth phase implies a complex process in which this pathogen interferes with the functions of the host cell, even controlling its own intracellular traffic. In this aspect it is known that when the expression of some proteins of macrophage are inhibited, the intracellular survival of *B. melitensis* is reduced in the early stages of phagocytosis [29]. Thus, when a smooth species of *Brucella* is phagocytized by the macrophage, it enters in an endocytic path, avoiding the hydrolytic degradation by fusion with the lysosomes, allowing its intracellular survival in the early stages of infection [30, 31]. Therefore, it is known that virulence of *Brucella* is associated with survival in phagocytic cells [25]. This could explain the results obtained by our research group, in which we observe a relationship between the reduction of the internalization of *B. melitensis* LVM31 mutant strain and a decrease in the survival and intracellular replication of the bacterium from 4 hours until the 72 hours post infection in both murine macrophages and HeLa cells. Based on these results, we conclude that Omp31 plays a key role in the invasion and intracellular multiplication capacity *of B. melitensis* Bm133[19].

Once we identified that the Omp31 protein plays an important role in the integrity of outer membrane properties and in the intracellular survival of *B. melitensis* in macrophages and epithelial cells, we further investigated what the effect of the mutation of *omp*31 would be on virulence of the bacterium in a murine model [19].

Although mice are not natural hosts of *Brucella* spp., they are the experimental laboratory model most used to study the virulence of *Brucella* in vivo [32]. In this model, *Brucella* can infect multiple tissues, including spleen and liver. During the chronic stages of infection, a recent study using bioluminescent *Brucella* strains found that bacteria target the salivary glands, which could be related to human infection, where inoculation occurs by ingestion of contaminated food. In addition, the mice presented a chronic infection of the tail joints with *Brucella* similar to osteoarticular brucellosis in animals and humans [33, 34]. The course of murine brucellosis depends on the virulence of the bacterial strain, the dose and the route of inoculation, as well as breed, genetic background, age, sex and physiological state of the mice. Therefore, more investigations are needed to clarify these experimental variables [32].

The replication profiles of the *Brucella* in spleen are highly reproducible and are developed in four phases: i), initiation or colonization of the spleen (first 48 h); ii), acute phase, from the third day until the moment when the bacteria reach maximum numbers; iii) chronic constant phase, where the bacterial numbers are plateaus; and iv), phase of chronic decline, during which bacteria of the genus *Brucella* are eliminated. This pattern shows clearly pathophysiological signs and is sensitive to small variations in virulence, which makes it possible to evaluate the attenuation when fully virulent bacteria are used as controls [23]. Based on these investigations we included different variables in the present study. We used two control strains of *Brucella melitensis* with different virulence degrees in order to determine the splenic colonization of *B. melitensis* LVM31 mutant strain in both male and female BALB/c mice compared to the control strains. Another variable that we included in the study was the physiological state of the animals, because in the present study we inoculated pregnant female mice with the different *Brucella* strains in order to determine the splenic colonization of the offspring since several studies have reported both vertical and horizontal transmission in offspring of experimentally infected females [35]. Therefore, we hypothesized whether the mutation of *omp*31 would cause a decrease in the propagation of LVM31 mutant strain through these transmission routes.

The results obtained in this work showed that the absence of the Omp31 protein caused a decrease in splenic colonization in both male and female Balb/c mice. In addition, organs of the mice inoculated with LVM31 mutant strain did not present lesions or apparent histopathological changes compared to mice inoculated with the control strains. These results are very similar to those obtained in other studies, where they demonstrated that the absence of this protein on outer membrane of the *B. ovis* PA strain, although it reduces by one logarithm the maximum levels of splenic colonization of the bacterium in mouse, it does not diminish its persistence in spleen [8, 36, 37].

Regarding the evaluation of the degree of propagation with the mutant strain in mice under study, our results showed that there was a decrease in splenic colonization in the mice born from the females inoculated with *B. melitensis* LVM31 mutant strain in comparison with the control strains.

These results could be explained because Omp31 has also been attributed an immunomodulatory role in *B. melitensis* 16M [38], as well as porin function characteristics [39] and of binding to heme groups in *B. ovis, B. melitensis* and *B. suis* [14]. However, it has also been found to have drastic effects on virulence in vivo of *B. melitensis* Rev1 and *B. ovis* PA, in the absence of Omp31 [8, 17]. According to data we compiled in our previous and present studies, we conclude that the Omp31 does is not only involved in maintaining the integrity of the outer membrane, and the intracellular survival of the bacteria in macrophages and epithelial cells [19], but that it also plays an important role on the virulence of *B. melitensis*, since all the experimental groups that were inoculated with *B. melitensis* LVM31 mutant strain showed a decrease in splenic colonization without lesions or apparent histopathological changes in spleen compared to the control strains.

Another significant aspect is the persistence of the bacteria on spleen, which is an indicator of virulence or attenuation, and is regularly used in the quality control test of vaccines (residual virulence). Vaccine candidates are often analyzed in mice by determining splenic colonization (CFU/ml count) after the challenge test with the appropriate virulent *Brucella* doses at the precise post-vaccination times. Since most live or killed *Brucella* vaccines provide some degree of protection in mice, controls in mice immunized with reference vaccines (S19 or Rev1) are critical. Finally, the mice have been used successfully to evaluate prophylactic or therapeutic applications against brucellosis [32].

Based on these investigations we set ourselves the objective of evaluating the protection conferred in mice immunized with the mutant strain (LVM31), the vaccine strain (Rev1) and PBS (negative control) against the challenge with an infective dose of *B. melitensis* Bm133 wild-type strain. The results obtained in the present work showed that there was a lower statistically significant persistence of bacteria in the spleen of mice immunized with LVM31 mutant strain and Rev1 vaccine strain compared with the mice immunized with PBS (*p* < 0.05), and in terms of the splenic colonization in the vaccinated mice with LVM31 mutant strain and Rev1 vaccine strain, there were no statistically significant differences at any time post challenge with the virulent strain. According with these results, we conclude that *B. melitensis* LVM31 conferred a protective response similar to *B. melitensis* Rev1 vaccine strain against the challenge with *B. melitensis* Bm133 virulent strain.

The results obtained in the present work allow us to conclude that Omp31 protein plays an important role in bacterial virulence because there was a significant decrease in the splenic colonization and a lower persistence of the bacteria (residual virulence) in the spleen of both male and female BALB / c mice experimentally infected with LVM31 mutant strain compared to the control strains.

Finally, because the mutation of *omp*31 caused a significant attenuation of the bacteria in both in vitro and in vivo assays, *B. melitensis* LVM31 mutant strain could be considered as a potential vaccine candidate for the control of goat and sheep brucellosis.

## Figures and Tables

**Table 1:** Bacterial strains.

Bacterial strain	Relevant genotype	Relevant characteristic	Origin
*Brucella melitensis* biotype 1 Bm133	Mexican reference strain (wild type)	Smooth virulent *Brucella* strain	MML^a^
*Brucella melitensis* Rev1	Vaccine reference strain	Smooth attenuated *Brucella* strain with a Streptomycin resistance	MML^a^
*Brucella melitensis* LVM31	*omp*31::Kan^r^ (mutant strain)	*Brucella melitensis* biotype 1 Bm133 with a Kanamycin resistance cassette inserted into the SalI site of *omp*31	Verdiguel *et al.* 2017 [19]

^a^MML, Molecular Microbiology Lab, Immunology and Microbiology Department, UNAM.

**Table 2:** Residual virulence determination in mice inoculated with *Brucella* strains.

Female		Male	
Group 1	10 female BALB/c mice inoculated with *B. melitensis* wild-type strain	Group 1	10 male BALB/c mice inoculated with *B. melitensis* wild-type strain
Group 2	10 female BALB/c mice inoculated with *B. melitensis* Rev1 vaccine strain	Group 2	10 male BALB/c mice inoculated with *B. melitensis* Rev1 vaccine strain
Group 3	10 female BALB/c mice inoculated with *B. melitensis* LVM31 mutant strain	Group 3	10 male BALB/c mice inoculated with *B. melitensis* LVM31 mutant strain
Group 4	10 female BALB/c mice inoculated with PBS	Group 4	10 male BALB/c mice inoculated with PBS

Female and male BALB/c mice were inoculated with approximately 5 × 10^5^ CFU of *B. melitensis* strains.

**Table 3:** Mice immunized with vaccine strains for evaluating the protection conferred against challenge of *B. melitensis* wild type.

Group 1	10 female BALB/C mice immunized with 1 × 10^9^ CFU of *B. melitensis* LVM31
Group 2	10 female BALB/C mice immunized with 1 × 10^9^ CFU of *B. melitensis* Rev1 vaccine strain
Group 3	10 female BALB/C mice immunized with PBS

**Table 4:** Corresponding groups for evaluating residual virulence in pups born of females inoculated with *Brucella* strains.

Group 1	10 female BALB/C mice inoculated with *B. melitensis* wild-type strain
Group 2	10 female BALB/C mice inoculated with *B. melitensis* Rev1 vaccine strain
Group 3	10 female BALB/C mice inoculated with *B. melitensis* LVM31 mutant strain
Group 4	10 female BALB/C mice inoculated with PBS
